# Exploring the Impact of Online Education on Scientific Presentation Skills in Women Neuroscience Students

**DOI:** 10.59390/001c.155318

**Published:** 2025-12-31

**Authors:** Stacey B. B. Dutton, Jennifer Larimore

**Affiliations:** 1 Agnes Scott College Neuroscience Department; 2 Neuroscience Department Agnes Scott College

**Keywords:** women in STEM, neuroscience education, remote learning, science communication, undergraduate students

## Abstract

Pedagogical shifts to online learning during the COVID-19 pandemic impacted learning outcomes for STEM students. Many courses have remained online after the pandemic, making it essential to assess the effectiveness of these practices on student skill development. While prior research has examined engagement, confidence, and self-efficacy, less is known about how online learning affects oral scientific communication skills. This study analyzed 23 undergraduate women enrolled in an upper-level neuroscience course at a women’s liberal arts college during Fall 2020. Using a pre/post format, we assessed students’ growth across three areas: (1) reading and analyzing primary neuroscience articles, (2) designing neuroscience-based experiments, and (3) developing and delivering oral scientific presentations. Assessments included weekly written article analyses and two oral presentations, evaluated with a rubric that measured experimental design, presentation slide quality, and oral communication. Results demonstrated significant improvement in students’ ability to critique scientific literature and construct professional presentation slides. In contrast, rubric scores for oral presentation performance showed no statistically significant gains, though this may reflect a ceiling effect, as most students scored at or near the rubric maximum in the pre-assessment. Taken together, these findings suggest that online learning environments can effectively support analytical and written aspects of science communication, while further refinement of assessment tools is needed to determine their impact on oral communication growth.

## INTRODUCTION

The COVID-19 pandemic led to a rapid shift in the method of course delivery across all academic institutions (Chu et al., 2025; Evans, 2022; Hamal & Aryal, 2022). The most common adaptation was the transition from in-person to online learning, using platforms such as Zoom. This immediate change created challenges for many instructors, as knowing how to properly and effectively deliver an online course was not part of their standard pedagogy. This shift was particularly complicated for those teaching STEM courses, as they rely heavily on active participation, discussions, and presentations to engage students with the course content. Of particular importance are science communication skills (both oral and written), as they are critical in neuroscience and scientific careers. However, it was unclear whether a virtual learning environment was adequate in supporting their development.

The importance of STEM students being able to effectively read, comprehend, and communicate scientific information is paramount. In classroom settings, primary research articles are an ideal tool for helping students understand the direct application of core science concepts and for supporting the development of effective written and oral communication skills. Traditional in-person formats are effective in fostering these skills by providing direct feedback, real-time interactions, and public speaking experiences. Though necessary at the time, online formats may alter how students engage with presentations, ultimately affecting their ability to cultivate these skills and impacting learning outcomes.

While our study takes place at an all-female liberal arts college, where women are not numerically minoritized, it remains critical to consider their experiences in the broader context of STEM. Women continue to be underrepresented in neuroscience and many related disciplines, and oral scientific communication skills are essential for their future participation in these male-dominated environments. Moreover, research suggests that women may face distinct barriers in online education (Corwin et al., 2021; Theobald et al., 2020), including lower engagement (Sung & Huang, 2024), reduced confidence (Landrum, 2020), and challenges in access to technology (McIntyre et al., 2025). These challenges can persist regardless of institutional setting and highlight the importance of examining how women adapt to virtual learning.

Previous studies have examined the impact of remote learning and have found benefits such as improved engagement and self-efficacy (Wang et al., 2022) and improved oral presentation construction in asynchronous formats compared to synchronous ones (Indriyani et al., 2024). However, limited studies have investigated how virtual formats affect oral scientific communication skills in STEM students, particularly among women at women’s colleges who, while not minoritized in their local classroom context, are preparing to enter broader STEM environments where gender imbalances remain the norm. This study analyzes pre- and post-assessment scores of students in an upper-level neuroscience course to determine whether they improve in their ability to analyze scientific articles, construct presentations, and deliver oral presentations. The findings from this study help inform strategies for strengthening science communication pedagogy in virtual settings, while also offering insight into how women in STEM navigate skill development in preparation for future professional contexts.

## MATERIALS AND METHODS

This study evaluates the effectiveness of remote learning in teaching core research communication and experimental design skills to undergraduate students during the COVID-19 pandemic in 2020. BIO 350 (*Cellular Neuroscience*) is an upper-level, research-focused neuroscience course that introduces students to core topics in cellular neuroscience, including: the history of neuroscience, research techniques, neuroanatomy, cellular composition of the brain, neuron cytology, neural development, membrane and action potentials, synaptic plasticity, neurotransmitters, receptors, and second messengers.

Throughout the course, we emphasized several NACE (National Association of Colleges and Employers) competencies across major assignments: Critical Thinking/Problem Solving, Oral Communication, Written Communication, Teamwork/Collaboration, Digital Technology, Research Skills, and Career Management. However, for this paper, we focus on the development of the following three competencies as demonstrated through the weekly *Article Presentations and Analysis* assignments: (1) *Critical Thinking/Problem Solving* – Through weekly article analyses, students critically read and evaluated scientific literature. They were also required to design follow-up experiments based on the articles, strengthening their ability to think critically about neuroscience concepts and research methodologies. (2) *Written Communication* – Students demonstrated scientific writing skills through the weekly analysis assignments and the development of their article presentation slides. (3) *Research Skills* – Students designed original experiments and communicated their work through both scientific writing and oral presentations. The following sections detail the course structure and methods used for data collection.

### Subjects

A total of 23 female participants were included in this study. Of these students, 19 self-identified as students of color. Specifically, 13 identified as Black or African American, 2 as Latina, and 4 as Asian, Asian American, or Pacific Islander (AAPI). The remaining 4 students identified as White. All participants were students enrolled at Agnes Scott College during the Fall 2020 semester. Due to the COVID-19 pandemic, all course content was delivered remotely via Zoom. The study was approved by the Agnes Scott College Institutional Review Board (IRB #B2024-25-02).

### Assignments

Over the semester, we assessed students’ competencies in problem-solving, scientific writing, and research experiment design through two key assignments: (1) group presentations of a primary research article and (2) weekly article analyses, which included an experimental design component. The sections below provide detailed descriptions of each assignment and the corresponding scoring criteria.

### Group Article Presentations

For this assignment, students were divided into groups of 2–3. Each week, one group presented a primary research article related to the course content discussed that week. Every member of the group was required to present data from the article and explain its significance to the overall hypothesis. Students were graded individually using a rubric (Supplementary Table) that assessed their ability to create slides that demonstrated professionalism and creativity, had an effective layout, and accurately conveyed the scientific content. In addition, students were evaluated on their oral presentation skills. Specifically, we assessed: (1) presentation style, defined by speaking pace, volume, and overall professionalism, and (2) presentation confidence, reflected in their command of and familiarity with the material.

Each criterion was scored on a scale from 1 to 3, with 1 indicating a low level of achievement and 3 representing the highest. Each group presented twice, once in the first half of the semester and again in the second, serving as pre- and post-assessments to measure improvement in these competencies over time.

### Weekly Article Analysis

**Figure 1.**
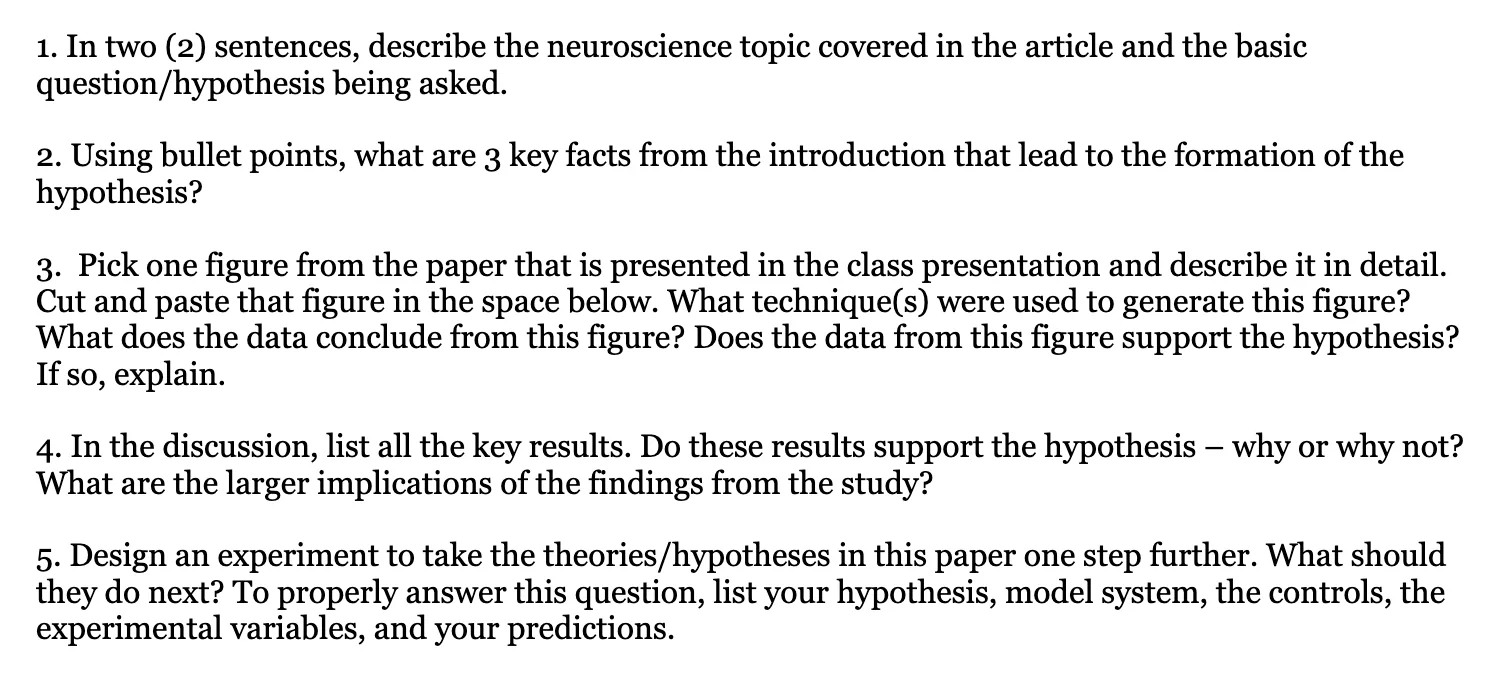
Article analysis questions. This figure represents the questions that students were required to respond to for the article analysis assignment. The assignment was designed to assess their comprehension of the article and their ability to construct a follow-up experiment.

For each assigned article, students completed a weekly article analysis assignment (Figure 1). This assignment was designed to develop scientific critical thinking and effective written communication skills.

Specifically, students were required to summarize relevant background information, identify the hypothesis, analyze one figure (including the associated methods and data), evaluate whether the figure supported the hypothesis, and assess the validity of the article’s conclusions. Additionally, students designed a hypothetical experiment to further investigate the findings of their assigned article. Each proposed experiment was required to include a hypothesis and/or research question, descriptions of experimental groups and controls, details on sample size and gender, an overview of the model system, a summary of methods, and a predicted outcome.

Students were evaluated on their ability to: (1) develop a clear experimental question or hypothesis, (2) select an appropriate experimental method to address the question or hypothesis, (3) discuss the analysis of the data, and (4) provide a well-developed predicted outcome. Each criterion was scored on a scale from 1 to 3, with 1 representing a low level of achievement and 3 representing the highest. A total of 10 assignments were used to evaluate students’ development in these competencies. The first and last assignments served as pre- and post-assessments to measure student progress.

### Instruction and Feedback

To prepare students for the first presentation, we incorporated several instructional scaffolds early in the semester. Students were introduced to strategies for reading primary literature, practiced analyzing figures in guided class exercises, and reviewed example slides from published neuroscience presentations. We also provided explicit instruction on experimental design, including how to formulate testable hypotheses, identify appropriate controls, and interpret expected outcomes. The grading rubric (Supplementary Table) was shared prior to the first presentation so that students could understand and self-assess the expectations for both written and oral components.

In addition to rubric scores, students received individualized written feedback on each assignment. For weekly article analyses, comments addressed clarity of scientific writing, accuracy of figure interpretation, and rigor of experimental design, with suggestions for refinement. For presentations, instructors provided qualitative feedback on slide organization, content accuracy, and clarity of explanation. Students were encouraged to revise and incorporate this feedback in subsequent assignments. Feedback was delivered primarily through written comments on submitted work and follow-up discussions during virtual office hours, which provided opportunities for clarification and targeted coaching.

### Statistical Analysis

To assess differences in students’ performance on the article presentation and article analysis between the pre- and post-tests, we calculated descriptive statistics (mean and standard error of the mean [SEM]) for each rubric category. Experimenters were blinded to all student identifiers. To determine statistical significance, we used the Wilcoxon signed-rank test. Statistical significance was defined as *p* ≤ 0.05, and error bars represent the SEM.

**Figure 2.**
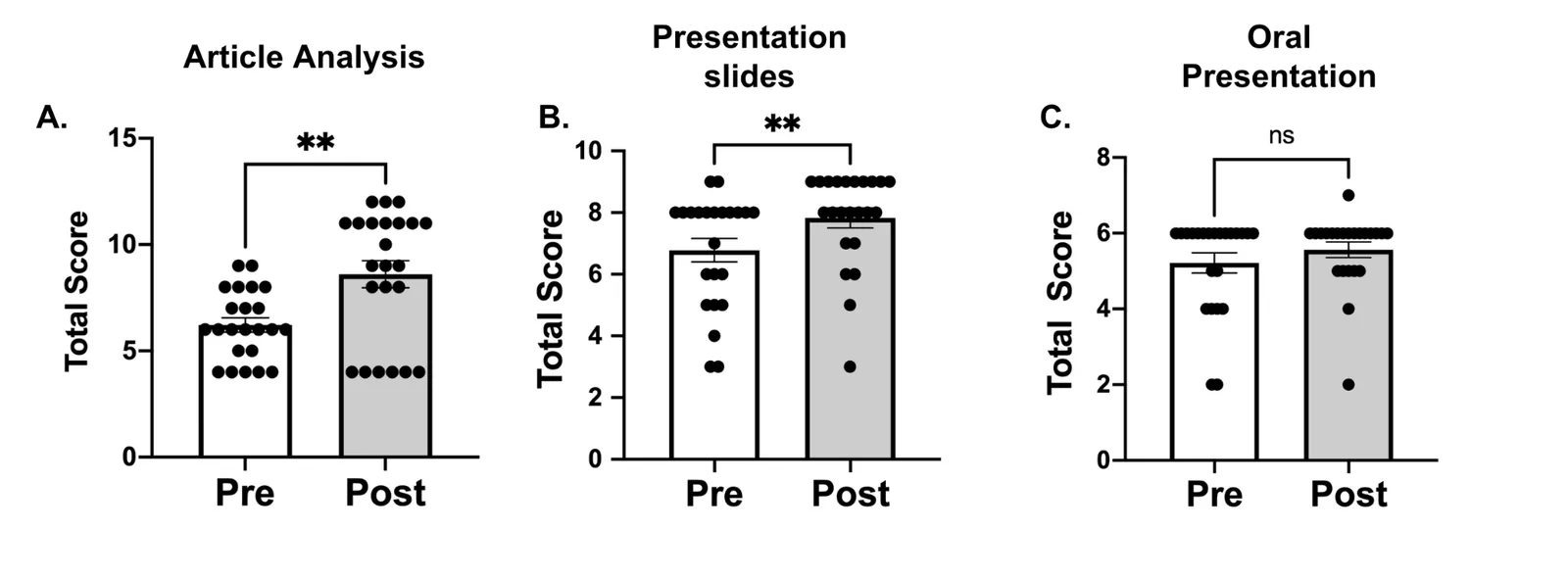
Students show improvement in their ability to comprehend primary articles and construct presentation slides, but not in oral presentation performance. This figure shows the total pre- and post-assessment scores for the components of the course: (*A) Article Analysis, (B) Presentation Slides, and (C) Oral Presentation*. Each dot represents an individual student’s score. Students demonstrated significant improvement in both article analysis and slide construction following the course. However, no significant improvement was observed in oral presentation skills (ns = not significant). ** represent (p ≤ 0.01). Error bars represent the standard error of the mean (SEM).

## RESULTS

### Students show improvements in article analysis and presentation slide construction, but not in oral presentation skills

Our primary areas of focus were (1) the students’ ability to read and analyze a primary research article, (2) their ability to construct professional presentation slides, and (3) their ability to effectively present a primary article in a virtual learning environment. Throughout the semester, students had two opportunities to present. The first presentation, delivered before midterms, served as a pre-test assessment, while the second, delivered later in the semester, served as the post-test. By the end of the semester, students showed significant improvement in their ability to read and critique a primary article (Figure 2A, Pre – 6.2 ± 0.3 vs. Post – 8.6 ± 0.6; p ≤ 0.01) and to create professional presentation slides (Figure 2B, Pre – 6.8 ± 0.4 vs. Post – 7.8 ± 0.3; p ≤ 0.01). For oral presentation performance, rubric scores did not indicate a statistically significant change (Figure 2C, Pre – 5.2 ± 0.3 vs. Post – 5.6 ± 0.2; ns). However, 17 of the 23 students scored at or near the maximum rubric value on the pre-assessment, suggesting a possible ceiling effect. This limitation may have obscured incremental improvements in delivery and confidence that were not captured by the three-point scale. Taken together, these findings suggest that while the virtual environment clearly supported growth in critical reading and slide construction skills, the assessment of oral communication requires more fine-grained tools to detect subtle gains over time.

**Figure 3.**
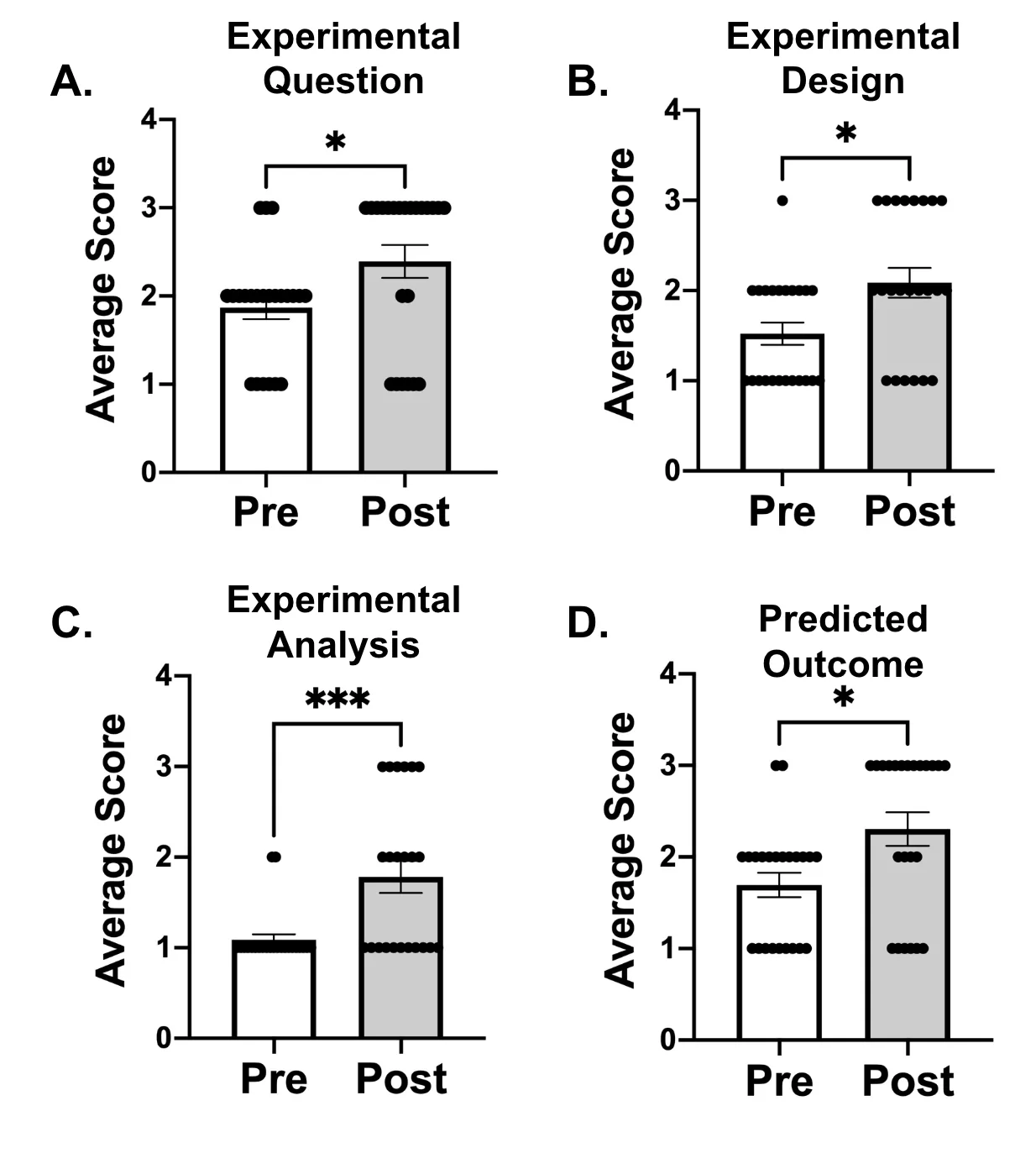
Student improvement in experimental design components. This figure shows average pre- and post-assessment scores for four components of the experimental design portion of the article analysis assignment: *(A) Experimental Question, (B) Experimental Design, (C) Experimental Analysis, and (D)* Predicted Outcome. Students showed significant improvement in all areas after course completion. Each dot represents an individual. * = p ≤ 0.05 and *** = p ≤ 0.001. Error bars represent the standard error of the mean (SEM).

### Improved performance in the article analysis assignment components

Students showed significant gains in their ability to analyze scientific articles within a virtual setting. To understand their areas of growth, we further evaluated specific components of the article analysis assignment.

We assessed students’ ability to design an experiment that built upon the findings of the selected primary article. In Figure 3A, we examined their ability to generate a well-defined research question or hypothesis. Students showed significant improvement in this area (Pre - 1.9 ± 0.1 vs. Post - 2.4 ± 0.2; p ≤ 0.05).

Next, we evaluated their ability to design an experiment using methods covered in the course (Figure 3B). Students again demonstrated significant gains (Pre - 1.5 ± 0.1 vs. Post - 2.1 ± 0.2; p ≤ 0.05). Their ability to select appropriate statistical analyses also improved (Figure 3C, Pre - 1.1 ± 0.1 vs. Post - 1.8 ± 0.2; p ≤ 0.001). Finally, their ability to predict outcomes based on their proposed experiment also improved (Figure 2D, Pre - 1.7 ± 0.1 vs. Post - 1.8 ± 0.2; p ≤ 0.05).

Together, these data indicate that students were able to effectively learn how to analyze scientific articles and propose follow-up experiments within the virtual learning environment.

**Figure 4.**
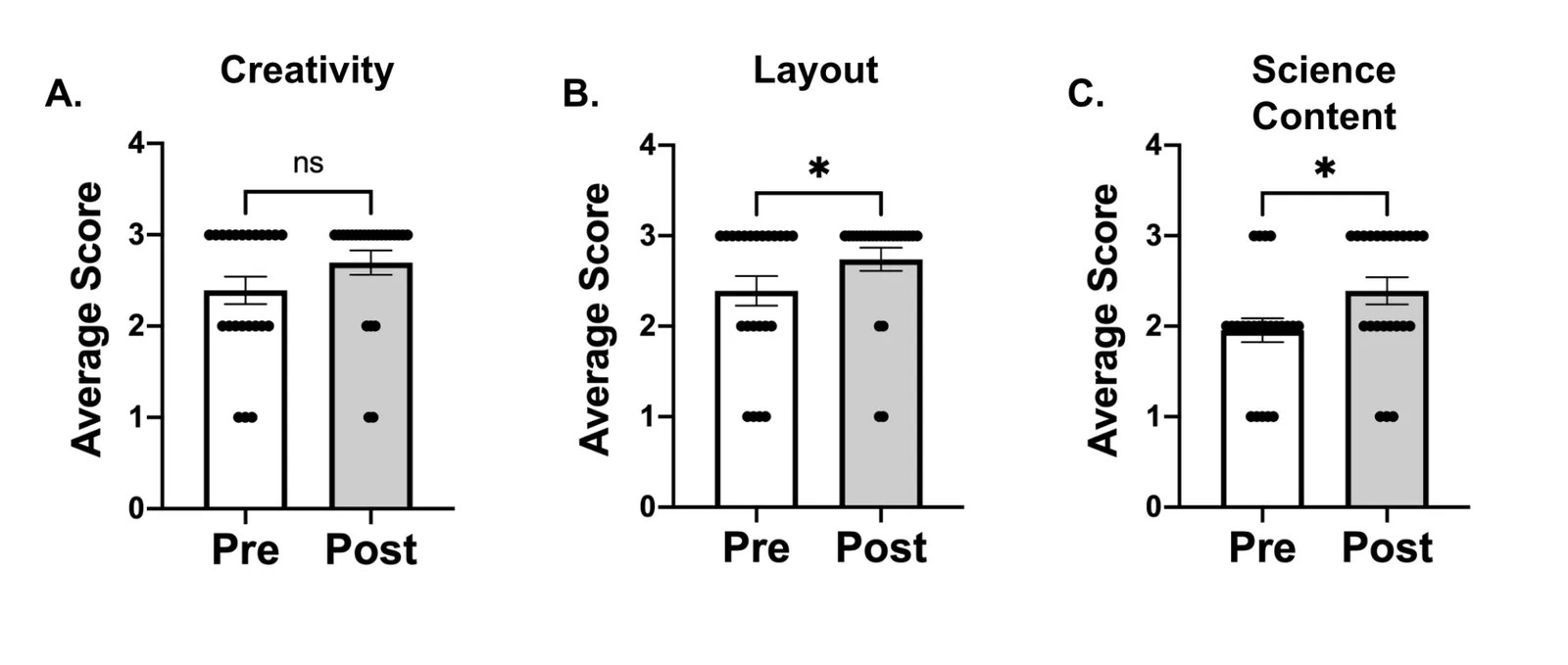
Students show improvement in some science presentation components. This figure displays average pre- and post-assessment scores for three components of students’ scientific presentation slides: (*A) Creativity, (B) Layout, and (C) Science Content*. Students showed significant improvement in layout and scientific content, while no significant change was observed in creativity (ns = not significant). Each dot represents an individual student’s score. * = p ≤ 0.05. Error bars represent the standard error of the mean (SEM).

### Students learned how to properly construct scientific presentation slides

Students demonstrated improvement in their ability to structure and design scientific presentation slides in a virtual setting (Figure 4). The components assessed included creativity (Figure 4A), layout design (Figure 4B), and scientific content (Figure 4C).

Students showed significant improvement in layout (Figure 4B, Pre - 2.4 ± 0.2 vs. Post - 2.7 ± 0.1; p ≤ 0.05) and in integrating appropriate scientific content (Figure 4C, Pre - 2.0 ± 0.1 vs. Post - 2.4 ± 0.2; p ≤ 0.05). However, there was no significant improvement in the creativity of the slides (Figure 4A, Pre - 2.4 ± 0.2 vs. Post - 2.7 ± 0.1; ns). These results suggest that students successfully adapted to designing visual aids for scientific communication in remote format.

**Figure 5.**
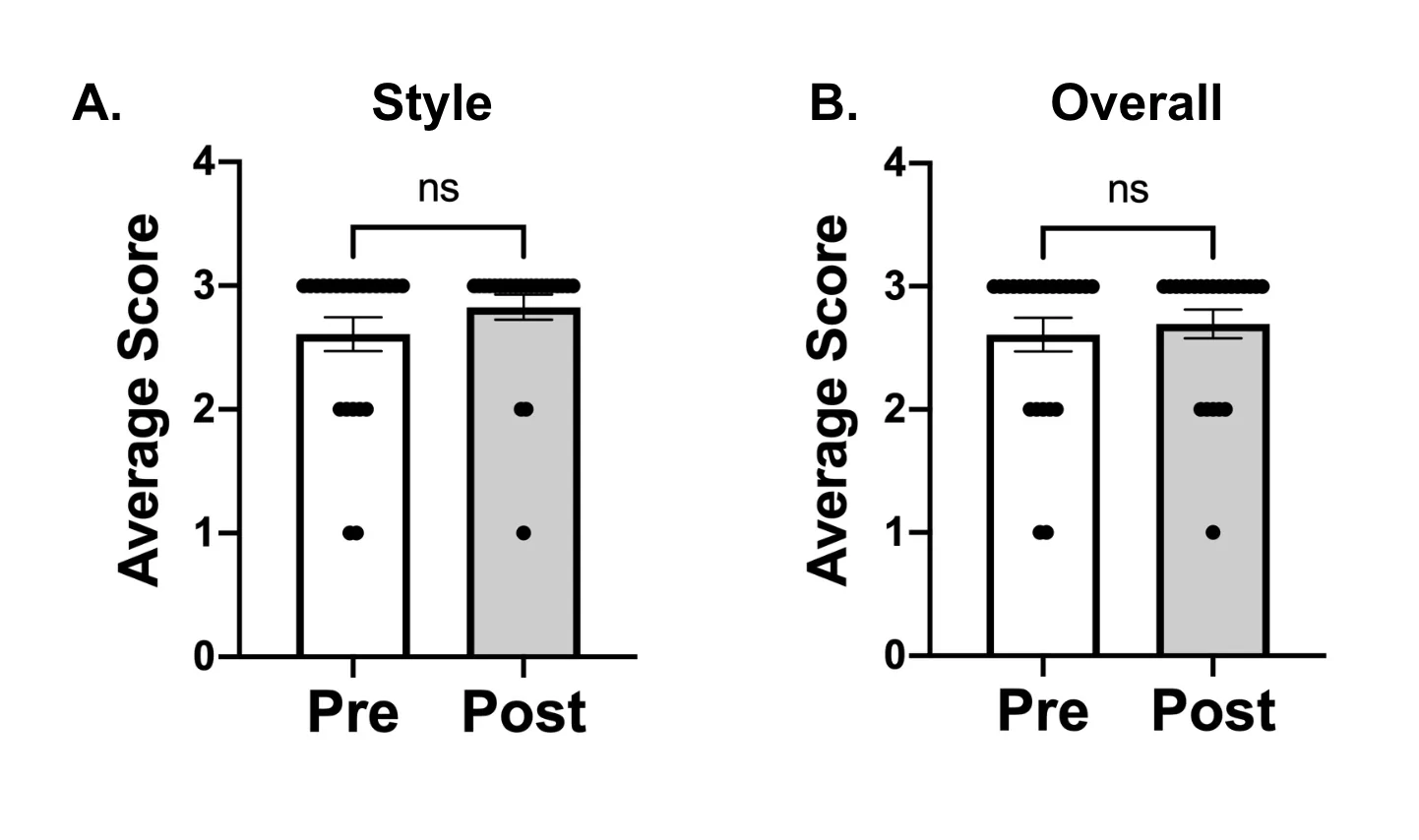
Students did not improve in oral presentation abilities. This figure shows average pre- and post-assessment scores for two aspects of students’ oral presentations: *(A) Presentation Style and (B) Overall Oral Communication*. No significant differences were observed between pre- and post-course scores in either category (ns = not significant). Each dot represents an individual student’s score. Error bars represent the standard error of the mean (SEM).

### Students did not improve their oral presentation skills in a virtual setting

Despite improvements in analysis and slide design, students did not show gains in oral presentation performance (Figure 5). Evaluations focused on presentation style (Figure 5A) and confidence (overall) (Figure 5B). No significant improvements were observed in either category (Figure 5A, Pre - 2.6 ± 0.1 vs. Post - 2.8 ± 0.1; ns; Figure 5B, Pre - 2.6 ± 0.1 vs. Post - 2.7 ± 0.1; ns). The scores for both categories were around 50% for the pre- and post-assessment, which may impact the outcomes. These findings suggest that the virtual environment may have limited opportunities for students to develop and practice strong oral communication skills

### DISCUSSION

In the current study, we sought to determine the impact of remote learning on the development of science communication skills in an upper-level neuroscience course. We found that the remote environment was effective in cultivating students’ abilities to analyze scientific articles, design neuroscience-based experiments, and construct effective presentation materials. These findings are consistent with previous studies suggesting that remote learning supports certain aspects of student performance. Wang et al. (2022) reported that students performed well in remote STEM courses, particularly in areas involving independent work. Similarly, Indriyani et al. (2024) found that students in remote learning environments produced higher-quality content, likely due to the additional time they could devote to refining their presentation materials and written work.

However, our data did not demonstrate enhanced growth in students’ oral communication skills. Students achieved an average score around 50% in both the pre and post assessments for oral presentations (Figure 5). While our rubric scores did not show significant growth in oral presentation skills, this result should be interpreted with caution. The majority of students scored at or near the rubric maximum in the pre-assessment, suggesting that a ceiling effect may have limited our ability to detect incremental improvements. Thus, while the virtual setting may indeed reduce opportunities for real-time feedback and peer modeling, it is also possible that the assessment tool lacked the sensitivity to capture more nuanced gains. Future studies should employ more fine-grained rubrics or multi-dimensional measures of oral communication (e.g., delivery style, scientific accuracy, audience engagement) to better evaluate student growth in this area. Additional studies are needed to determine what aspects of remote learning contributed to this lack of growth and to compare these results with in-person versions of the same assignments.

While our study provides quantitative evidence of skill development in analytical and visual communication, it does not capture students’ perspectives on why oral communication skills showed limited growth. Factors such as technology challenges, reduced engagement in virtual settings, presentation anxiety, or “Zoom fatigue” may have contributed to this outcome. Future research would benefit from incorporating qualitative methods, such as interviews or focus groups, to explore student perceptions of barriers and supports. These insights could inform the development of targeted pedagogical strategies, such as integrating practice opportunities, peer feedback structures, and wellness supports, that address both the technical and affective dimensions of oral scientific communication in online settings.

Our findings also highlight the importance of considering how feedback and modeling differ between virtual and in-person learning contexts. In the remote environment, students primarily received feedback in the form of written rubric comments and individualized notes on their article analyses and presentation slides. While this structured, detailed feedback likely contributed to the measurable improvements in slide design and experimental analysis, it could not fully replicate the dynamic modeling and spontaneous corrective feedback present in in-person classrooms.

Traditionally, in-person instruction allows students to observe multiple peer presentations early in the semester, participate in live question-and-answer exchanges, and receive immediate verbal guidance from the instructor. These experiences provide real-time modeling of effective communication strategies and help normalize the process of public speaking in front of an audience. In contrast, in the virtual setting, students presented directly to a Zoom screen with limited opportunities for peer modeling or interactive feedback. This difference may explain why analytical and visual communication skills improved, while oral presentation skills remained stagnant.

By clarifying the instructional scaffolds and feedback approaches in our course, our results contribute to the pedagogical literature on supporting STEM students’ communication skills. Specifically, they suggest that written and structured feedback can be effective for improving scientific writing, experimental design, and slide construction in virtual environments. However, the absence of live modeling and interactive engagement limits growth in oral scientific communication. Future course designs should consider incorporating opportunities for synchronous peer feedback, small-group breakout discussions, or recorded practice presentations with instructor commentary to better approximate the benefits of in-person modeling.

Another factor that may contribute to the limited growth in oral communication skills is the absence or distortion of key visual and auditory cues in online environments. Prior work has shown that the loss of micro-expressions, eye contact, and subtle audience reactions can significantly disrupt communicative feedback loops during videoconferencing, leading speakers to experience uncertainty about audience engagement and reducing their ability to adjust pacing or tone effectively (Fauville, 2023). Similarly, Li et al. (2024) reported that frequent videoconferencing can lead to communication and information overload, both of which contribute to videoconference fatigue. These findings suggest that online learning imposes not only perceptual barriers (e.g., limited nonverbal feedback and muted audience response) but also cognitive burdens that tax students’ attentional resources. When combined, these factors may constrain students’ ability to refine oral communication skills, as cognitive load is redirected toward managing technology and interpreting fragmented feedback rather than toward improving delivery or audience awareness. Consequently, while our rubric scores did not reveal measurable improvement in oral presentation performance, it is plausible that these technological and cognitive constraints masked more nuanced growth that was not captured by the current assessment tool.

It is important to note that this study was conducted at a women’s liberal arts college, where female students are not numerically minoritized in the classroom. The absence of gender imbalances such as stereotype threat or instructor bias toward male students, removes some of the systemic barriers often reported in co-educational STEM contexts. This makes our findings particularly noteworthy: even in a supportive environment where gender equity is structurally embedded, the online format still limited students’ development of oral scientific communication skills. These results suggest that the challenges of building confidence and oral communication proficiency in virtual environments may be even more pronounced in co-educational or male-dominated STEM settings, where additional systemic barriers exist.

Taken together, these results highlight the importance of intentional design in remote STEM pedagogy. Oral scientific communication is a core competency for success in STEM careers, and it requires opportunities for practice, immediate feedback, and audience interaction (Martin & Bolliger, 2018). In-person learning often provides these experiences through class discussions, peer feedback, and live presentations, whereas online environments can reduce the richness of these interactions (Sung & Huang, 2024; Ventura Roque-Hernández et al., 2024). Our findings underscore the need for instructors to design online courses with built-in opportunities for authentic audience engagement, structured peer interaction, and supportive feedback to build communication confidence.

In summary, our study shows that remote learning can effectively support the development of analytical and technical skills but may fall short in promoting oral communication growth, even in an environment that eliminates certain gender-based inequities. As such, remote STEM course design must account not only for content delivery but also for the intentional cultivation of inclusive and interactive structures that prepare women to thrive as communicators in the broader, male-dominated STEM landscape.

### Address correspondence to:

Stacey B. B. Dutton, PhD, Department of Biology and Neuroscience, Agnes Scott College, 141 E. College Avenue

Decatur, GA 30030. Email: sdutton@agnesscott.edu

## Supplementary Material

Supplementary Table
