# Supplementary material for: Exploring the Impact of Online Education on Scientific Presentation Skills in Women Neuroscience Students
Source: J Undergrad Neurosci Educ. 2025 Dec 31;24(1):75–82. doi: 10.59390/001c.155318 (PMC12867018; doi:10.59390/001c.155318)
Supplement: Supplementary Table [file junejournal_2025_24_1_155318_324842.pdf]

Criteria	High (3)	Medium (2)	Low (1)
**Experimenal Desgn (AA)**	***Experiments Purpose:*** The reseach question of problem/ hypothesis is well-defined and connected to the prior knowledge of the study	***Experiments Purpose:*** The question to the problem/ hypothesis is defined adequatley, but lacks a clear rationale or purpose that stems from the content of the study	***Experiments Purpose:*** The research question/ hypothesis is not clearly defined, lacks purpose and/or rationale, and/or does not connect to the content of the study
**Date of assignement**	***Design:*** The experimental design is appropriate to the problem. The important varables are clearly defined and include mention of the gender (if animals), sample size, and all approapriate controls. Methods explicilty stated.	***Design:*** The design is appropriate to the problem, but fails to explain important variable(s) and/or adequate controls or conditions. Methods maybe be the best for the proposed experiment.	***Design:*** The design is inappropriate to the problem. It fails to explain important variable(s) and/or adequate controls or conditions. Specific methods are not stated.
	Data Anaylsis: Mentions correct stats for analysis of data	***Data Anaylsis:*** Mentions limited information for data analysis and/or some incorrect statistical methods	***Data Anaylsis:*** Does not mention specific statistical methods for analyzing the data
	Expected Outcome(s): Makes speculations on the outcome of the proposed experiment based on their understandings of the paper and class content	***Expected Outcome(s):*** Makes some speculations on the outcome of the proposed experiment based on their understandings of the paper, but may not connect to relevant evidence and to the class content	***Expected Outcome(s):*** Does not make any specualtions on the outcome of the experiments
**SCORE =**			
**Article Presentation**	***Overall Asethic/Creativity:*** The presentation has a conhesive theme that uses appropriate fonts, colors, and graphs	***Overall Asethic/Creativity:*** The presentation has an visually appealing, but there are issues with either the fonts, colors, and/or graphs.	***Overall Asethic/Creativity:*** The presentation consist of mostly text and appears hastily assembled.
**Date of assignement**	***Layout:*** Figures/Graphs/ Tables are presented in a clear and consistently. Presents a story that is well organized and appropriate. Each slide with data clearly provides an explanation of purpose, results, and connection to hypothesis or goal of the study.	***Layout:*** Figures/Graphs/ Tables are organized in an appropriate way, but some places may lack clarity or consistency. Ther may also be extraneous materials. Data slides maybe inconsistent at providing the purpose, results, and connection to hypothesis or goal of the study.	***Layout:*** Little evidence of a cohesive plan for the layout and design. Jumbled and/or disconnected. Data slides do not provide the purpose, results, and connection to hypothesis or goal of the study.
	Science Content: The relevant science content is presented and is provided in enough details for clear understanding. Presents a story that is well organized and appropriate.	***Science Content:*** The relevant science content is presented and is sometimes provided in enough details for clear understanding. Overall story may not be consistently clear.	***Science Content:*** The relevant science content is not presented and/or not in enough details for clear understanding. There is no overall story.
**SCORE =**			
**Oral Presentation**	***Presentation Style:*** Good speaking pace, volume, and eye contact. Well reshearsed.	***Presentation Style:*** Overall, an adequate style but may have some issues with speaking pace, volume, and eye contact.	***Presentation Style:*** Overall, inadequate style with major issues with speaking pace, volume, and eye contact.
**Date of assignement**	***Presentation Confidence***: Demonstrated a command of the materials and was able to answer the questions with clear, thoughtful explanantions that are based on the concepts of the paper or class themes. Shows a mastery of the content	***Presentation Confidence***: Demonstrated a knowledge of the materials, but may lack the ability to make connections to the larger themes of the course.	***Presentation Confidence***: Demonstrated a lack of knowledge of the materials and the inability to make connections to the larger themes of the course.
**SCORE =**			

Supplementary Table. Grading rubric for article analysis and presentation assignments. This table presents the rubric used to evaluate students' performance on the article analysis, presentation, and oral communication components of the assignment. The rubric was designed to assess student understanding of experimental design, scientific content, data interpretation, and communication skills. Each criterion is rated on a three-point scale (High, Medium, Low) to provide detailed feedback on both written and oral competencies in a virtual neuroscience learning environment.
